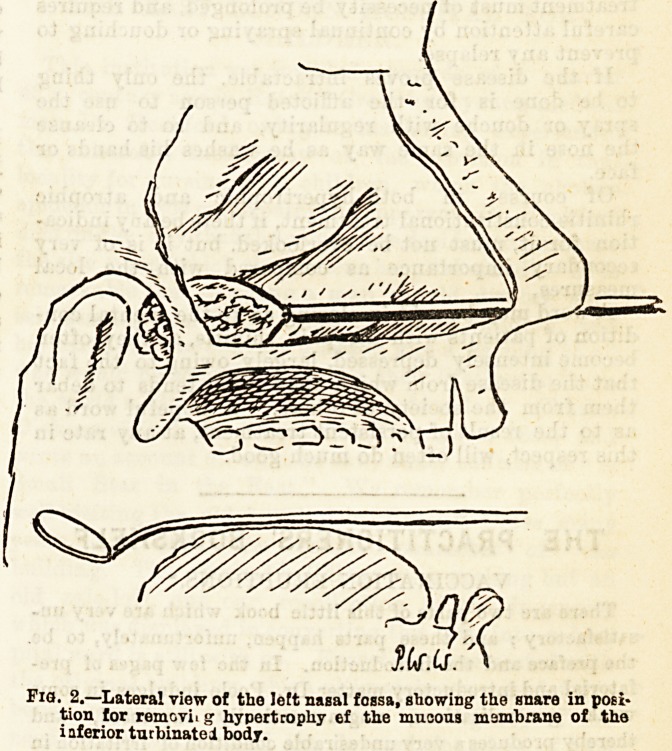# The Treatment of Rhinitis

**Published:** 1893-04-29

**Authors:** 


### CENTRAL LONDON THROAT HOSPITAL.

### The Treatment of Rhinitis.

Rhinitis, or inflammation of the nasal cavities, may be divided into three kinds, viz., acute, chronic hypertrophic, and chronic atrophic.

Of the first kind, which is known more commonly under the popular name of "a cold in the head," we do not propose to speak, as it naturally does not often present itself for treatment at a hospital, but the two latter varieties of rhinitis make up a large proportion of the cases at the Central London Throat Hospital.

First, then, as to *Hypertrophic Rhinitis.* The most common complaint in this disease is either of a constant discharge from the nostrils, which escapes anteriorly or into the naso-pharynx, or of nasal obstruction.

In the slightest cases, where the inferior turbinated bodies are but little enlarged, the line of treatment adopted is merely with a view to dealing with the flow of serum and mucus, and for this end some pulvis potassii chloratis co. is given, which consists of chlorate of potash, ½ oz.; borax, ½ oz.; bicarbonate of soda, ½ oz.; white sugar, 1 oz. The directions given with it are: Let a measured teaspoonful be dissolved in a quarter of a pint (half a tumbler) of tepid water. Half the solution to be gently injected with a syringe along the floor of each nostril night and morning. After use blow the nose freely. Along with this is prescribed unguentum eucalypti (olei eucalypti m xx., vaseline ℥i), a little of which is to be put inside each nostril after the injection. It may be incidentally mentioned here that the nasal douche is now practically given up at the Central London, except in cases of atrophic catarrh, and for this reason, that if there be any obstruction to the return of the fluid along the opposite nostril, there is not a little danger that some of the fluid may be forced through the eustachian tubes into the middle ear, perhaps setting up acute otitis media as a result.

Returning, however, to the treatment of hypertrophic rhinitis; very many of the cases require more effectual and energetic treatment than the use of lotion and

Fig. 1.—Transverse section showing well-marked hypertrophic rhinitis, causing thickening and hypertrophy of the middle and inferior turbinated bodies.—(Zuckerkandl).

ointment. Such cases are those in which the obstruction to nasal respiration is the main symptom, and this is due to an enlargement of the inferior turbinated body, which enlargement may be general, or located more at its anterior or posterior part. There are three ways in which this enlargement is generally dealt with according to circumstances, namely, by the application of caustics, the use of the snare (with the wire either cold or hot), or by the use of the galvano-cautery point.

The caustic used is almost always chromic acid, and it is applied in the following way. The nasal cavity is first cocainized either by spraying it, or more commonly by plugging the cavity with cotton wool soaked in a twenty per cent. solution of the drug. The effect of the anæsthetic is to much reduce the size of the hypertrophied body owing to the contraction of blood vessels, which it causes. The cavity is then well exposed by a speculum, the surface dried with cotton wool applied on the end of a copper probe with a screw end, the other end of which is flattened, and has fused on to one side of the flattened part the solid chromic acid, the side of the probe next the septum being kept perfectly clean, to prevent it cauterising the septum if it should perchance touch it. The chromic acid on the probe is now applied flatly to the turbinated body. Should the septum be injured, the danger is that a bridge of union may form between it and the turbinal. After a few minutes the effect of the acid is neutralised by a little alkaline solution. After a few days, when the slough is loose, it should be removed with a pair of forceps, and a little pulv. pot. chloratis co. given for use as above. Removal of the slough greatly diminishes the discomfort of the patient. The snare is very frequently used in these cases, that in most favour is the cold snare. Of the different varieties in vogue we cannot enter into the merits here, but any form in which the hand is permitted to be out of the line of sight of the operator, and in which the wire works fairly smoothly, will serve. The kind of cases in which it is specially used are those in which the turbinal has undergone a somewhat polypoid degeneration, and so can easily be enclosed in the wire loop through an anterior speculum; or, again, those cases in which the posterior end of the body is enlarged and projects into the naso-pharynx. The difficulty here is often great to get the mass enclosed and the loop, and the method adopted is to pass a finger through the mouth behind the palate into the naso-pharynx, and so by the aid of touch to manipulate the wire into position. This however, requires a good deal of dexterity. Should the galvanic cautery snare be used, the wire must only be heated to a dull redness. It is rarely, however, necessary, as the bleeding is not often formidable by use of the other methods. The galvano-cautery burner on the other hand, is in frequent use, either in the form of the flat burner, or the point. To use it the nostril is cocainised and dried out, then Lennox Browne's speculum (which has ivory blades, and so does not get heated), is introduced. The point is then introduced cold, heated and then thrust horizontally backward, and in this way made to pierce the hypertrophied tissue in one or two places. If the flat burner be used this is made hot while it is applied to the surface of the mucus membrane. Just as in the case of the chromic acid, the greatest care must, of course, be taken not to touch the septum with the hot point. The subsequent treatment is the same as if acid had been used. There is a fourth means of removing the enlargement, which, however, we believe is not so largely employed as the others. This consists in shaving off the projecting part by means of Carmalt Jones's instrument which cuts like a plane or spoke-shave. In the hands of some this is found to be quite efficient. It must here be mentioned that in using the cautery in the nose the greatest gentleness and care must be exercised, as cases have been observed in which either from want of care or using a too high temperature, acute suppurative catarrh of the middle ear has been set up, and also empyema of the antrum.

One additional reason for treating the enlarged turbinal when it causes nasal obstruction may be here mentioned, namely, that by hindering ventilation of the middle ear, the obstruction is a not unfrequent cause of deafness.

*Atrophic Rhinitis* is, perhaps, more familiarly known by one of its symptoms, which makes it a most distressing disease—namely, ozæna, though this may be due to other causes, such as syphilitic and tubercular disease of the nose. This, however, is the symptom for which patients apply for treatment, and it is to the removal of this most abominable affliction that endeavours are largely directed. The most important thing here is cleanliness and attention to the complete removal of the scanty discharge of foul-smelling mucus, which forms crusts in the nostrils, and produces the odour, while assisting in keeping up the disease. It is generally the custom to begin the treatment in the out-patient room, by a complete removal of all crusts. This may be done in one or two different ways. A common way is to stuff both nostrils as full as possible with cotton wool through a speculum by the aid of a pair of angled forceps. The effect of this is to so irritate the membrane lining the nose, that fluid is poured out, which loosens the crusts, which can then be removed with ease through a speculum by the forceps. Great care must be taken, if the crusts adhere firmly, not to cause bleeding, as this does harm. Another method very commonly used is to wash out the nose with the nasal coarse spray, using in it a solution consisting of borax gr. viij., bicarbonate of soda gr. viij., carbolic acid gr. iij., water ℥j. (Dobell's solution). In using this spıay the patient is seated, breathing through his mouth, with a bowl to catch the fluid under his chin. The nozzle to the spray is then fitted to one nostril, and the ball squeezed; the fluid then passes in at one side and out at the other, just as with the nasal douche. To thoroughly cleanse the posterior part of the nose a nozzle with a bent end can be attached, which is passed into the naso-pharynx behind the soft palate. After the spray, the nose must be carefully examined to see that no crusts are left. The patient is then advised as to use of the spray night and morning, and ordered the solution. Instead of this spray the pulv. potassii chloratis is sometimes

FIG. 2.—Lateral view of the left nasal fossa, showing the snare in position for removi[illegible]g hypertrophy of the mucous membrane of the inferior turbinated body.

ordered to be used as described above. In addition to this he is ordered an oily solution of an antiseptic to be used after the spray, so as to prevent the formation of crusts. The best of these is, perhaps, the solution of menthol ʒss. in olive oil, ℥i. to be used in an oily spray-producer. Other preparations are iodol or eucalyptol used in the same proportion in the same way, or an ointment consisting of oil of eucalyptus mxx., iodol gr.v., vaseline ℥j. If this treatment be persisted in, it can scarcely be said that a cure will result, but the condition of the patient will be such that he can go into society without being an intolerable nuisance to those around him. There is, however, another way of treating these patients in addition to the above, which is believed by some not only to alleviate the symptoms of the disease, but to present some hope of its cure. The principle of this form of treatment is to cause sufficient irritation and stimulation of the mucous membrane, to change the atrophic process and improve the condition of the mucous membrane. There are several methods employed, and with some of them it is necessary in order to get proper supervision and carrying out of the treatment to take the patient into the hospital for a time.

The one most commonly used in the out-patient department is, we believe, to give the patient some iodoform wool, with directions. in addition to the spray, &c., to stuff one of the nostrils alternately as full as he can with the wool, and if he is able to sleep with it so all night. Another way is to paint the whole of the inside of the nose (after it has been cleansed) with a ten per cent. solution of trichlor-acetic acid, or with diluted tincture of iodine 1-7. Then, again, a small roll of Canthos paper may be used in the following way: After the nostril has been thoroughly cleansed as usual a piece of this paper is cut about an inch square and made into a roll, which is introduced into the nostril (only one is treated at a time), and left there as long as the patient will allow, which varies from about twenty minutes to perhaps an hour or two, and then removed. This may be repeated at intervals of about a fortnight, as, indeed, may the treatment by painting the nostril. Whichever method be used the treatment must of necessity be prolonged, and requires careful attention by continual spraying or douching to prevent any relapse.

If the disease proves intractable, the only thing to be done is for the afflicted person to use the spray or douche with regularity, and so to cleanse the nose in the same way as he washes his hands or face.

Of course. in both hypertrophic and atrophic rhinitis constitutional treatment, if there be any indication for it, must not be overlooked. but it is of very secondary importance as compared with the local measures.

A word may be said in closing as to the mental condition of patients with atrophic rhinitis, as they often become intensely depressed, largely owing to the fact that the disease from which they suffer tends to debar them from the society of others. A hopeful word as as to the result of persistent treatment, at any rate in this respect, will often do much good.

## Figures and Tables

**Fig. 1. f1:**
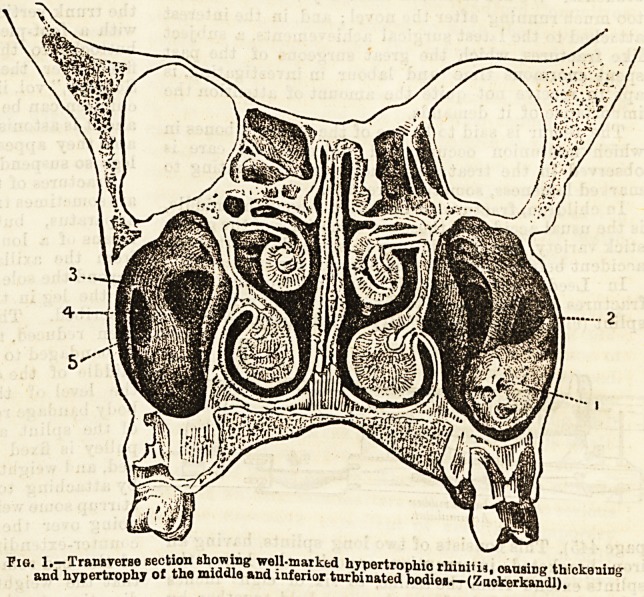


**Fig. 2. f2:**